# Targeted therapy combined with local consolidative surgery for oligometastatic stage IVA lung adenocarcinoma with CCDC6-RET fusion: a case report

**DOI:** 10.3389/fonc.2026.1811218

**Published:** 2026-05-20

**Authors:** Faxian Gao, Yijun Fan, Qingchun Fan, Tingrui Mei, Muhammad Quais Khan, Aier Zhou, Hangyu Liu, Jingsi Dong

**Affiliations:** 1 Department of Thoracic Surgery, West China Hospital, Sichuan University, Chengdu, Sichuan, China; 2 Lung Cancer Center/Lung Cancer Institute, West China Hospital, Sichuan University, Chengdu, Sichuan, China; 3 West China School of Medicine, Sichuan University, Chengdu, Sichuan, China; 4 Department of Outpatient Department, West China Hospital, Sichuan University, Chengdu, Sichuan, China

**Keywords:** CCDC6-RET fusion, local consolidative therapy, non-small cell lung cancer, oligometastatic, selpercatinib

## Abstract

**Background:**

Precision therapy for non-small cell lung cancer (NSCLC) has expanded to include rare driver genes. RET fusion is a rare target in lung cancer, and data on targeted therapy combined with local consolidation surgery in advanced patients with oligometastatic status are limited. This article reports a case of successful surgical resection following initial systemic treatment with selpercatinib.

**Case presentation:**

A 68-year-old never-smoking male was diagnosed with adenocarcinoma of the right upper lobe (cT3N3M1, stage IVA) with ipsilateral pulmonary metastasis and cervical and axillary lymph node metastases, consistent with oligometastatic features. A CCDC6-RET fusion was identified via next-generation sequencing. The patient received selpercatinib for three months as initial systemic therapy.

**Results:**

Post-treatment imaging assessment indicated a partial response. The patient subsequently underwent successful thoracoscopic anterior segmentectomy of the right upper lobe with lymph node dissection. Postoperative pathology confirmed 15% residual viable tumor cells, which did not achieve major pathological response (MPR). Due to marked shrinkage and scar-like changes in the originally enlarged lymph nodes after targeted therapy, limited lymph node dissection was performed intraoperatively, and all submitted lymph nodes were negative. The postoperative pathological stage was ypT1aN0M0. Adjuvant selpercatinib was continued postoperatively, with no signs of recurrence during 7 months of follow-up.

**Conclusion:**

This case demonstrates that for selected patients with advanced lung adenocarcinoma harboring a CCDC6-RET fusion and oligometastatic disease, initial systemic therapy with selpercatinib followed by local consolidative surgery is a feasible multimodal strategy. This is not a routine recommendation for stage IV lung cancer but requires individualized decision-making after multidisciplinary discussion in specific oligometastatic cases.

## Introduction

Lung cancer is the leading cause of cancer-related mortality worldwide, with NSCLC constituting the majority of cases (1). The development of precision medicine has significantly improved outcomes for advanced-stage patients through targeted therapies against common driver genes such as EGFR and ALK. However, a subset of patients harbor rare genetic alterations, including RET, ROS1, and NTRK fusions. RET fusions occur in approximately 1-2% of NSCLC cases and are more common in young, never-smoking patients with adenocarcinoma (2, 3). Patients with stage IV NSCLC have traditionally been considered ineligible for surgery, even when the disease is initially unresectable. However, with the introduction of the oligometastatic concept and improvements in systemic therapy, for oligometastatic patients without distant organ metastasis, reassessing local treatment (surgery or stereotactic body radiotherapy) after effective initial systemic therapy has become a worthwhile multidisciplinary approach. This article reports a case of stage IVA lung adenocarcinoma with a CCDC6-RET fusion and oligometastatic features that achieved significant pathological regression and underwent successful surgery following initial systemic therapy with selpercatinib, aiming to inform treatment strategies for this patient subgroup.

## Case presentation

### Patient information and history

A 68-year-old Asian male presented with a 5-month history of dry cough. He had no smoking history or family history of cancer. Physical examination revealed enlarged right supraclavicular lymph nodes.

### Initial diagnosis and baseline assessment

#### Imaging

**Figure 1:**
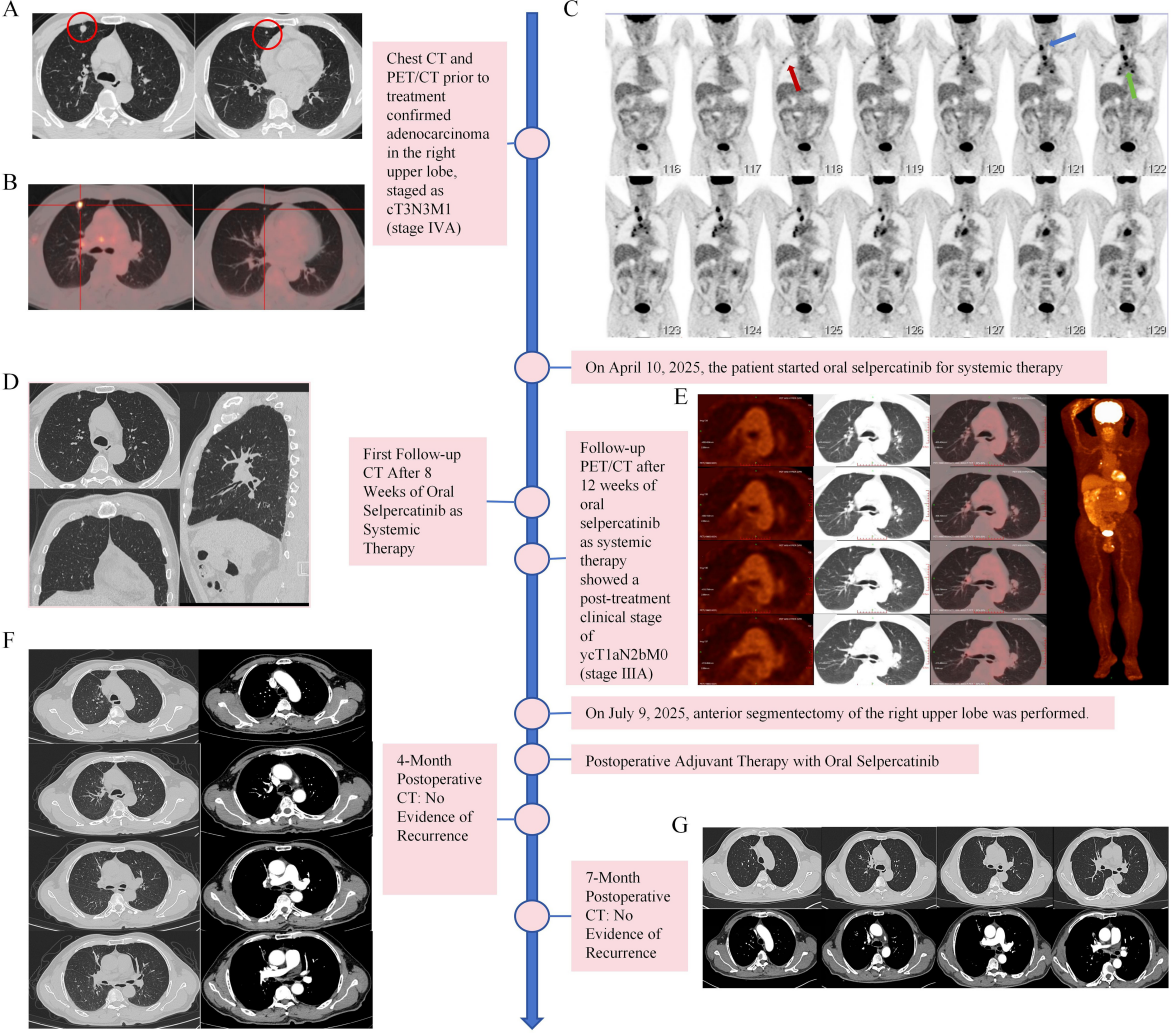
Treatment workflow and imaging evaluation before and after treatment. **(A)** Baseline chest CT shows a solid nodule in the anterior segment of the right upper lobe (maximum diameter approximately 1.2 cm) in the left panel, and another solid nodule within the same lobe (size approximately 0.5 cm × 0.4 cm) in the right panel. **(B)** Baseline PET-CT shows increased FDG uptake in the anterior segment nodule of the right upper lobe (SUVmax: 7.56), with another solid nodule identified in the same lobe. **(C)** Baseline PET-CT shows increased FDG uptake in multiple lymph nodes in the right supraclavicular region (blue arrow), right axilla (red arrow), and mediastinum (green arrow), with an SUVmax of 9.01 in station 2R. **(D)** CT scan after 8 weeks of selpercatinib treatment shows marked reduction in the primary lesion. **(E)** PET-CT after 12 weeks of treatment shows the primary lesion reduced to 0.7 cm × 0.8 cm with no increased FDG uptake; no lymph nodes with increased FDG uptake are observed in the right axilla or supraclavicular region. **(F)** Chest CT at 4 months post-surgery shows no signs of recurrence. **(G)** Chest CT at 7 months post-surgery still shows no signs of recurrence.

Chest CT showed a solid nodule (approximately 1.2 cm × 1.1 cm) in the anterior segment of the right upper lobe (**Figure 1A**), another solid nodule (0.5 cm × 0.4 cm) in the same segment, and enlarged hilar and mediastinal lymph nodes (stations 2R, 4R, 4L, 7, 10R), with the largest measuring 1.9 cm × 2.9 cm. PET-CT revealed a poorly defined, irregular nodular soft tissue density in the anterior segment of the right upper lobe with spiculation and lobulation, abutting the adjacent pleura, showing increased FDG uptake (SUVmax: 7.56, SUVavg: 5.54) (**Figure 1B**). Multiple enlarged lymph nodes with increased FDG uptake were noted in the right hilum, mediastinum (stations 2R, 4R, 4L, 7, 10R), right axilla, and the right supraclavicular region. The 2R station lesion had an SUVmax of 9.01 (SUVavg: 4.48), measuring approximately 2.3 × 1.9 cm at its largest dimension (**Figure 1C**). Thyroid ultrasound showed no significant abnormalities. Whole-body imaging assessment revealed no distant organ metastases to the liver, bones, brain, or other organs.

#### Physical examination

Enlarged right supraclavicular lymph nodes were palpated. The thyroid was not enlarged, non-tender, and without vascular bruits.

#### Clinical stage

According to the 8th edition TNM staging system, the diagnosis was adenocarcinoma of the right upper lobe (1.2 cm × 1.1 cm) with ipsilateral pulmonary metastasis (0.5 cm × 0.4 cm) and lymph node metastases in the hilum, mediastinum, right neck, and right axilla, resulting in a clinical stage of cT3N3M1, stage IVA.

#### Pathological diagnosis

Pathological examination of a right supraclavicular lymph node needle biopsy revealed atypical cells, consistent with metastatic lung adenocarcinoma based on immunohistochemistry.

#### Molecular pathology

Next-generation sequencing of a blood sample, covering target-related genes and fusions or rearrangements (EGFR, MET, ALK, ROS1, KRAS, BRAF, ERBB2, NTRK, etc.), identified a RET mutation (CCDC6-RET fusion). Additionally, PD-L1 was highly expressed, with a tumor proportion score (TPS) of 80%. No other driver gene alterations, including EGFR, ALK, ROS1, BRAF, and KRAS, were detected.

### Multidisciplinary team discussion and treatment decision

Following multidisciplinary discussions involving the departments of Thoracic Surgery, Medical Oncology, Radiology, and Pathology at the hospital, the patient was diagnosed with stage IVA lung cancer, with metastatic lymph nodes confined to the supraclavicular and axillary regions and no evidence of distant organ metastasis, which met the criteria for oligometastatic disease (OMD). The patient had an acceptable performance status at the time of initial treatment (ECOG 1), with no major vascular or tracheal invasion. Although stage IV lung cancer is generally not recommended for curative surgery, in the oligometastatic setting, if initial systemic therapy can achieve significant downstaging, subsequent local treatment may be considered. Given the presence of a RET fusion, and despite high PD-L1 expression, driver gene-positive tumors generally respond poorly to immunotherapy alone, consistent with previous studies showing low response rates to immunotherapy in patients with RET fusion-positive NSCLC (4). Referring to the high objective response rate (ORR 84%) of selpercatinib in advanced RET fusion-positive lung cancer from studies such as LIBRETTO-431 (5), and taking into account the patient and his family’s reluctance to receive chemotherapy or immunotherapy, the multidisciplinary team decided to initiate conversion therapy with selpercatinib (160 mg orally twice daily).

### Treatment course and response evaluation

The patient received selpercatinib for 3 months (12 weeks). Treatment-related adverse events included mild diarrhea and rash, managed symptomatically without any grade ≥3 events. CT re-evaluation at 8 weeks (**Figure 1D**) showed significant shrinkage of both the primary lesion and enlarged lymph nodes. Repeat PET-CT at 12 weeks, assessed according to RECIST 1.1 criteria, confirmed a partial response with a 33% reduction in the sum of target lesion diameters. The primary lesion shrank to 0.7 cm × 0.8 cm with no increased ¹^8^F-FDG uptake; no lymph nodes with increased ¹^8^F-FDG uptake were observed in the right axilla or right supraclavicular region; several lymph nodes in the right hilum and subcarinal region showed abnormally increased ¹^8^F-FDG uptake (maximum SUV 9.67), with corresponding slightly high density, the largest measuring approximately 11 mm × 7 mm. The post-treatment imaging stage was ycT1aN2bM0, stage IIIA (**Figure 1E**). Given the significant treatment response and the persistence of suspicious hypermetabolic lymph nodes, the MDT re-evaluated and decided to attempt local surgical treatment.

### Surgery and postoperative recovery

Following initial systemic therapy, the patient underwent uniportal thoracoscopic anterior segmentectomy of the right upper lobe with lymph node dissection. The procedure was uneventful, without intraoperative complications.

### Postoperative pathological assessment

#### Gross specimen

A nodule measuring 0.8 cm × 0.7 cm × 0.6 cm was identified adjacent to the pleura, well-demarcated from surrounding lung tissue. The cut surface was gray-white to gray-red, solid, and firm.

#### Microscopy

**Figure 2:**
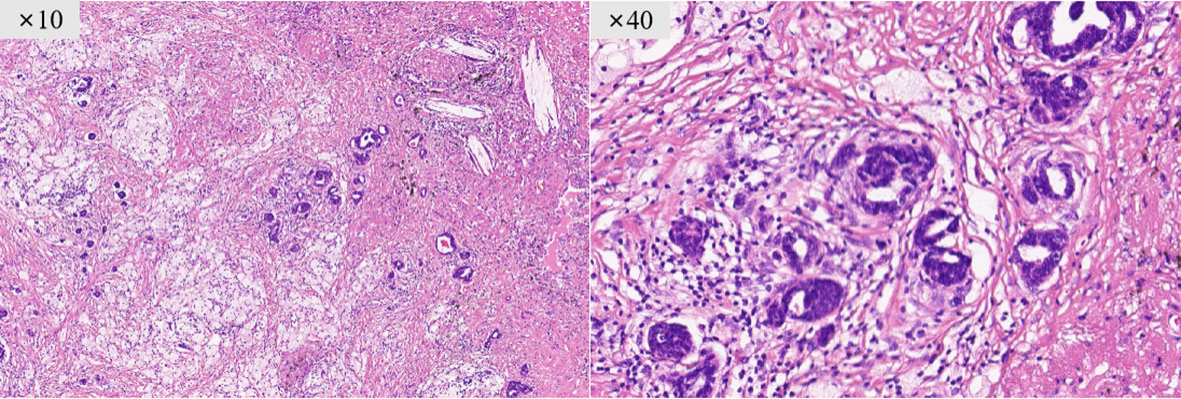
Postoperative pathological findings. The primary lesion shows extensive fibrosis and histiocytic infiltration, with scattered residual viable adenocarcinoma cells (predominantly acinar pattern). The proportion of residual viable tumor cells is approximately 15% (H&E staining).

Tumor was classified as invasive non-mucinous adenocarcinoma, predominantly of the acinar pattern, with moderate differentiation. Extensive fibrosis and histiocytic infiltration were observed within the primary lesion, with scattered residual viable adenocarcinoma cells. The proportion of residual viable tumor cells was estimated to be approximately 15%, which did not reach major pathological response (MPR) (**Figure 2**).

#### Lymph node status

Due to the significant shrinkage or scar-like changes of the originally enlarged lymph nodes after targeted therapy, only 3 stations with a total of 4 lymph nodes were dissected intraoperatively, all of which were negative for malignancy (0/4). The specific stations dissected included stations 2R, 4R, and 7.

#### Postoperative pathological stage

Based on the postoperative pathology, the primary lesion showed significant regression (ypT1a), and all dissected lymph nodes were negative (pN0). Therefore, the final postoperative pathological stage was ypT1aN0M0.

### Postoperative adjuvant therapy and follow-up

Based on the good response to systemic therapy and pathological downstaging, and after discussion with the patient, adjuvant therapy with oral selpercatinib (same dosage) was continued. Regular chest CT scans were scheduled every 3–6 months. A chest CT at 4 months post-surgery showed no signs of recurrence (**Figure 1F**). At the latest follow-up (7 months post-surgery), the patient showed no evidence of disease recurrence (**Figure 1G**), maintained a good quality of life, and had an ECOG performance status of 0.

## Discussion

This case illustrates the successful use of initial systemic therapy with selpercatinib in a patient with stage IVA CCDC6-RET fusion-positive lung adenocarcinoma with oligometastatic features, achieving a partial response and followed by local consolidative surgery. Its clinical significance lies in providing an alternative multidisciplinary treatment approach beyond traditional palliative care for this rare subgroup of oligometastatic patients.

For stage IV lung cancer with distant organ metastasis, conversion to surgical status via any systemic therapy is generally not recommended, because even if the local lesion is resected, potential micrometastases will lead to rapid recurrence. In contrast, at initial diagnosis, our patient had only extraregional lymph node metastases (supraclavicular, axillary) without liver, bone, brain, or other hematogenous distant metastases, falling within the oligometastatic spectrum. Accumulating evidence indicates that in oligometastatic NSCLC, radical local treatment (surgery or stereotactic radiotherapy) to the primary and metastatic sites can significantly improve progression-free and overall survival (6, 7). Some guidelines now explicitly state that for oligometastatic NSCLC, after effective initial systemic therapy, local radical treatment to all lesions should be considered (8). In this case of oligometastatic stage IVA CCDC6-RET fusion-positive lung adenocarcinoma, after effective initial systemic therapy, local surgical resection was performed following multidisciplinary discussion.

In this case, selpercatinib induced 67% radiographic tumor regression. Postoperative pathology confirmed a significant treatment response (15% residual viable tumor cells), though major pathological response (MPR) was not achieved, demonstrating the efficacy of the initial targeted therapy. The postoperative pathological negativity of the lymph nodes suggests that selpercatinib was also effective against cervical and axillary metastases.

The rationale for the treatment strategy in this case centers on the clear advantages of targeted therapy and the limitations of immunotherapy. First, the phase III LIBRETTO-431 study demonstrated that first-line treatment with selpercatinib achieved a median progression-free survival (PFS) of 24.8 months in patients with RET fusion-positive advanced non-small cell lung cancer (NSCLC), significantly superior to that in the chemotherapy-plus-immunotherapy group (11.2 months; hazard ratio, 0.46), with objective response rates of 84% and 65%, respectively (5). This comparison directly confirms the superiority of RET-targeted therapy over immunotherapy-containing regimens. Second, although the patient in this case had a PD-L1 tumor proportion score (TPS) as high as 80%, the multicenter RET-MAP study showed that patients with RET fusion-positive tumors receiving immunotherapy alone achieved a median PFS of only 3.1 months, substantially lower than the 16.2 months observed with RET inhibitors (4). Real-world studies have further confirmed poor responses to immunotherapy in patients with RET fusions (9), with some case reports indicating a risk of hyperprogression (10). From a biological mechanism perspective, RET fusion-positive tumors typically exhibit a low tumor mutational burden and a relatively “cold” immune microenvironment, which may underlie their poor response to immune checkpoint inhibitors (11, 12). Therefore, in the presence of a clearly targetable driver alteration (RET fusion), prioritizing highly effective and low-toxicity targeted therapy has become a fundamental principle of precision medicine. Additionally, the patient’s performance status was somewhat compromised at the initial diagnosis, and the patient expressed reluctance to receive chemotherapy or immunotherapy. Following multidisciplinary team (MDT) discussion, selpercatinib was selected as the first-line conversion therapy.

Regarding the extent of lymph node dissection, it is important to note that following targeted therapy, the originally enlarged lymph nodes had markedly regressed or even formed scar-like tissue. Consequently, during systematic lymph node dissection, it was challenging to accurately identify and completely dissect the previously positive nodal stations, resulting in a limited number of lymph nodes harvested. This represents an objective limitation of surgery following targeted therapy and warrants full disclosure in the present case. Despite the limited number of lymph nodes dissected, all submitted specimens were negative for malignancy, which, together with the significant pathological regression observed in the primary tumor, further supports the efficacy of selpercatinib.

While selpercatinib has shown remarkable efficacy in advanced RET fusion-positive NSCLC (5), its role in multimodal treatment for oligometastatic disease remains exploratory. Our case provides positive early practical evidence for its use in this setting. The optimal duration of postoperative adjuvant therapy is currently undefined, and future studies are needed to determine the optimal treatment duration.

The limitations of this report include its nature as a single case report, meaning that this treatment model should not be widely adopted for routine stage IV lung cancer. Instead, local consolidative surgery may only be considered on an individualized basis after thorough multidisciplinary discussion in strictly selected patients with oligometastatic disease (without distant organ metastasis) who have achieved a good response to systemic therapy. Furthermore, the postoperative follow-up period in this case is only 7 months, and long-term survival benefits require extended observation. Additionally, resistance mutation testing was not performed on the residual tumor cells, and the risk of clonal evolution under continued targeted therapy pressure warrants vigilance.

## Conclusion

This case demonstrates that for selected patients with advanced lung adenocarcinoma harboring a CCDC6-RET fusion and oligometastatic disease, initial systemic therapy with selpercatinib followed by local consolidative surgery is a feasible multimodal strategy. This approach can significantly reduce tumor burden, achieve deep pathological response, and thereby achieve long-term disease control within the oligometastatic framework. This underscores the critical importance of comprehensive molecular testing in advanced NSCLC to guide individualized, multidisciplinary treatment decisions.

## Data Availability

The original contributions presented in the study are included in the article/supplementary material. Further inquiries can be directed to the corresponding author.
